# Independent and combined effects of sleep quality and night sleep duration on health-related quality of life in rural areas: a large-scale cross-sectional study

**DOI:** 10.1186/s12955-022-01936-8

**Published:** 2022-02-21

**Authors:** Wei Liao, Xiaotian Liu, Ning Kang, Lulu Wang, Zhihan Zhai, Jing Yang, Xueyan Wu, Yongxia Mei, Shengxiang Sang, Chongjian Wang, Yuqian Li

**Affiliations:** 1 Department of Epidemiology and Biostatistics, College of Public Health, Zhengzhou University, 100 Kexue Avenue, Zhengzhou, 450001 Henan People’s Republic of China; 2 Department of Clinical Pharmacology, School of Pharmaceutical Science, Zhengzhou University, Zhengzhou, Henan People’s Republic of China

**Keywords:** Sleep quality, Night sleep duration, HRQoL, Rural area

## Abstract

**Background:**

The combined effect of sleep quality and night sleep duration on health-related quality of life (HRQoL) remains unclear, especially in resource-limited countries and areas. This study aimed to explore the independent and combined effects of sleep quality and night sleep duration on HRQoL.

**Methods:**

A total of 21,926 eligible participants from the Henan rural cohort study were selected. The Pittsburgh Sleep Quality Index was utilized to evaluate sleep quality and night sleep duration. The Tobit regression model, generalized linear model (GLM), and logistic regression model were performed to assess the associations of sleep quality and night sleep duration with HRQoL. The restricted cubic spline was applied to identify the dose–response relationships of sleep quality and night sleep duration with HRQoL.

**Results:**

After multivariable adjustment, the Tobit regression and GLM indicated that the regression coefficients [95% confidence interval (*CI*)] for poor sleep quality were − 0.124 (− 0.133, − 0.114) and − 6.25 (− 6.71, − 5.78) on utility index and VAS score, respectively. Compared with the reference group (7 h-), participants with short sleep duration (< 6 h) or long sleep duration (≥10 h) reported a lower HRQoL. A U-shape relationship between night sleep duration and HRQoL was observed, along with a J-shape relationship between sleep quality and HRQoL (*P* for non-linear < 0.001). Furthermore, individuals with longer night sleep duration (≥10 h) and poorer sleep quality were strongly associated with lower HRQoL (utility index [odds ratio (OR) (95% *CI*)]: 6.626 (3.548, 8.920), VAS score [OR (95% *CI*)]: 2.962 (1.916, 4.578)).

**Conclusion:**

Poor sleep quality and extreme night sleep duration were independently and combinedly associated with low HRQoL, suggesting that maintaining good sleep quality and appropriate night sleep duration was important.

*Clinical Trial Registration*: The Henan Rural Cohort Study has been registered at Chinese Clinical Trial Register (Registration number: ChiCTR-OOC-15006699). Date of registration: 06 July, 2015. http://www.chictr.org.cn/showproj.aspx?proj=11375.

**Supplementary Information:**

The online version contains supplementary material available at 10.1186/s12955-022-01936-8.

## Background

Sleep disturbance mainly includes insomnia, narcolepsy, obstructive sleep apnea, and restless legs syndrome, which have adverse effects on health status [1–4]. These sleep disturbances are usually characterized by poor sleep quality and abnormal sleep duration. Previous studies have reported that poor sleep quality and extreme sleep duration (both short and long) are associated with a broad spectrum of health outcomes, such as cognitive impairment, depressive symptoms, anxiety, coronary heart diseases (CHD), hypertension, and all-cause mortality [5–10].

Health-related quality of life (HRQoL), a multidimensional concept consisting of physical health status and subjective satisfaction with health, is a reliable indicator to assess health status [11]. Prior studies have indicated that some chronic conditions (including dyslipidemia, hypertension, depression, stroke, heart disease, and cognitive dysfunction) were associated with low HRQoL [12–15]. However, limited evidence is available on the associations of sleep quality or sleep duration with HRQoL.

Two previous studies have investigated the independent associations between sleep quality or sleep duration and HRQoL in older Chinese adults [16, 17]. These studies find that poor sleep quality and extreme sleep duration (both short and long) are associated with low HRQoL. In addition, three previous studies conducted in the US, Spain, and Korea also find a U-shape association between sleep duration and HRQoL [18–20]. However, the combined effect of sleep quality and night sleep duration on HRQoL remains unclear, especially in resource-limited countries and areas.

Therefore, the current study was conducted to investigate the independent and combined effect of sleep quality and night sleep duration on HRQoL among the Chinese rural population.

## Methods

### Study population

The current study was embedded in the Henan rural cohort study, which was a population-based study with a large sample of rural people living in Yuzhou, Suiping, Tongxu, Xinxiang, and Yima counties of Henan province in China. The cohort began in 2015 when 39,259 participants aged from 18 to 79 were included with a response rate of 93.7%. Detailed information of the cohort has been described elsewhere [21].

In the current study, a total of 23,559 participants were invited to the European Quality of Life Five Dimension Five Level Scale (EQ-5D-5L) questionnaire survey. Individuals with completed information on EQ-5D-5L, sleep quality, and night sleep duration (n = 23,328) were included. Considering the effect of shift work on sleep quality and night sleep duration, we excluded 1402 participants with shift work in the past 6 months. Finally, 21,926 eligible participants were included.

The Henan Rural Cohort Study was approved by the Zhengzhou University Life Science Ethics Committee and conducted following the principles of the Declaration of Helsinki (Code: [2015] MEC (S128)). Before the study commenced, participants were informed of the study’s purpose, health benefits, and potential hazards. Participants were required to provide informed consent, and both the researchers and respondents agreed to use the data for scientific research purposes only.

### Data collection

A structured questionnaire, including information on demographic characteristics, lifestyle factors, and personal history of chronic diseases, was conducted by well-trained research staff according to a face-to-face interview. Demographic variables included gender, age in years (18–44, 45–54, 55–64, and 65–79), marital status (married/cohabiting, widowed/divorced/separated, and single), educational level (elementary school or below, junior high school, and senior high school or above), and average monthly income (< 500 RMB, 500 RMB, and ≥ 1000 RMB). Lifestyle factors included smoking, alcohol drinking, and physical activity. Individuals who smoked at least one cigarette per day for six sequential or cumulative months were defined as current smokers. Those who consumed alcohol at least 12 times per year were considered as current drinkers. According to the validated Chinese version of the International Physical Activity Questionnaire (IPAQ) [22], physical activity was classified as low, moderate, and high. Chronic diseases, which included hypertension, dyslipidemia, type 2 diabetes mellitus (T2DM), coronary heart disease (CHD), and stroke, were collected through physical examination, laboratory tests, or self-reported physician diagnosis. The height and weight of participants were measured twice, and the average readings were computed to analyze. Body mass index (BMI, kg/m^2^) was calculated as weight (kg) divided by the square of height (m).

### Definition of sleep quality and night sleep duration

Sleep quality and night sleep duration were calculated according to the validated Chinese version of the Pittsburgh Sleep Quality Index (PSQI) over the previous month [23, 24]. The score of PSQI was calculated as the sum of the six components scores of PSQI (including subjective sleep quality, sleep latency, habitual sleep efficiency, sleep disturbance, use of sleep medication, and daytime dysfunction). PSQI score > 5 was identified as poor sleep quality, while PSQI ≤ 5 was identified as good sleep quality. Participants were asked two questions during the interview: “What time did you usually go to bed at night and How long can you fall asleep?” and “What time did you usually wake up in the morning?”. Night sleep duration was defined as the time interval between falling asleep and waking up in the morning. The night sleep duration was divided into six groups: < 6 h, 6 h-, 7 h-, 8 h-, 9 h-, and ≥10  h.

### Assessment of HRQoL

The EQ-5D-5L, consisting of two parts, was a tool widely used to measure HRQoL, especially for people with lower education levels and the elderly [25]. In the current study, a validated Chinese version EQ-5D-5L instrument was performed to assess the HRQoL of participants [26]. The first part of the EQ-5D-5L instrument consisted of five dimensions, including mobility (MO), self-care (SC), usual activities (UA), pain/discomfort (PD), and anxiety/depression (AD). Each dimension consisted of five levels (no problems, slight problems, moderate problems, severe problems, and extreme problems). The respondents were asked to choose one of five options for five dimensions, each of which best describes his/her health status on the day of the interview. According to the recently available Chinese value set for the EQ-5D-5L instrument [27], the EQ-5D-5L utility index was calculated by the formula as follows:

$${\text{Utility}} = {1} - {\text{MO}} \times {\text{L}}_{{\text{n}}} - {\text{SC}} \times {\text{L}}_{{\text{n}}} - {\text{UA}} \times {\text{L}}_{{\text{n}}} - {\text{PD}} \times {\text{L}}_{{\text{n}}} - {\text{AD}} \times {\text{L}}_{{\text{n}}} \;\left( {{\text{n}} = {1},{ 2},{ 3},{ 4},{ 5}} \right).$$

The utility index ranged from − 0.391 to 1.000, with 1 representing full health, 0 representing death, and a score < 0 representing a health status worse than death. The utility index was further classified as a binary variable with the cutoff value 1. Full health was identified as utility index = 1, while the low utility index was identified as utility index < 1 [28].

The EQ-5D-5L also included a visual analogue scale (VAS), a vertical 0 to the 100-point rating scale, reflecting the degree of satisfaction with their health status. The best and worst health states carry a score of 100 and 0, respectively. The VAS score was also classified as a binary variable with a cutoff value of 80 [28]. While the low VAS score was defined as the VAS score less than 80, the percentage of low VAS score was 34.35% which was similar to the low utility index (31.25%). In addition, to our knowledge, most Chinese consider a score above 80 as a good mark. Thus, we classified the VAS score as a binary variable with the cutoff value of 80 as face valid for the population.

### Statistical analysis

The statistical description was presented as mean with standard deviation (SD) for continuous variables and frequency with percentages for categorical variables. T-test or Kruskal–Wallis test was performed to compare differences between different groups for continuous variables, while Chi-square test was utilized for categorical variables.

The multivariate Tobit regression model [29] was used to investigate the association between sleep quality and night sleep duration and utility index because the distribution of the EQ-5D utility was skewed, and the utility index was censored at 1. Due to the VAS score being abnormal distribution, the generalized linear model (GLM) was used. Model 1 was unadjusted. Model 2 was adjusted age, gender, marital status, education level, average monthly income, physical activity, smoking status, drinking status, napping, and BMI. Model 3 was further adjusted for common chronic diseases based on model 2 (including hypertension, dyslipidemia, T2DM, CHD, and stroke). The associations of sleep quality and night sleep duration with utility index and VAS score were examined by binary logistic regression analyses. To identify the dose–response association of sleep quality and night sleep duration with HRQoL, we used the restricted cubic spline [30], where the reference point was 5 score of sleep quality and 7 h of night sleep duration.

Data were analyzed using SPSS 23.0 software package (SPSS Institute, Chicago) and STATA 15 for Windows. All *P* values were two-tailed with a statistical significance level of 0.05.

## Results

### Characteristics of study participants

Characteristics of study participants stratified by sleep quality are presented in Table 1. A total of 4606 individuals were defined as having poor sleep quality with a prevalence of 21.00%. The mean age ± SD of participants (39.47% male and 60.53% female) was 55.93 ± 12.44. Compared with the good sleep quality group, the poor sleep quality group was more likely to be older, female, had lower education level, lower average monthly income, and less physical activity, and less likely to be current smoker, current drinker, married, and had napping habit (all *P* < 0.05). In addition, the poor sleep quality group was more likely to suffer from chronic diseases. The difference between the good and poor sleep quality group in BMI was non-statistical significance. The mean (SD) utility index of the total sample, good sleep quality group, and poor sleep quality group were 0.923 (0.113), 0.964 (0.094), and 0.909(0.160), respectively. Besides, the mean (SD) VAS scores of the total sample, good sleep quality group, and poor sleep quality group were 78.09 (14.90), 79.69 (14.08), and 72.10 (16.33), respectively. Participants with good sleep quality had a higher utility index and VAS score than those with poor sleep quality (*P* < 0.001). The characteristics of study participants stratified by night sleep duration are listed in Additional file 1: Table S1. The differences in all the selected variables between different night sleep duration groups were statistically significant (all *P* < 0.001).

**Table 1:** Characteristics of study participants according to sleep quality

Variable	Total (n = 21,926)	Good (n = 17,320)	Poor (n = 4606)	*P*
Age (year, mean ± SD)	55.93 ± 12.44	55.16 ± 12.74	58.81 ± 10.81	< 0.001
Women n (%)	13,272 (60.53)	9974 (57.59)	3298 (71.60)	< 0.001
Educational level n (%)				
Elementary school or below	9834 (44.85)	7324 (42.29)	2510 (54.49)	< 0.001
Junior high school	8192 (37.36)	6657 (38.43)	1535 (33.33)	
Senior high school or above	3900 (17.79)	3339 (19.28)	561 (12.18)	
Marital status n (%)				
Married/cohabiting	19,714 (89.91)	15,694 (90.61)	4020 (87.28)	< 0.001
Widowed/separated/divorced	1856 (8.46)	1323 (7.64)	533 (11.57)	
Single	356 (1.62)	303 (1.75)	53 (1.15)	
Average monthly income n (%)				
< 500 RMB	8376 (38.20)	6373 (36.80)	2002 (43.46)	< 0.001
500- RMB	6961 (31.75)	5549 (32.04)	1412 (30.66)	
≥ 1000 RMB	6589 (30.05)	5397 (31.16)	1192 (25.88)	
Physical activity n (%)				
Low	7510 (34.25)	5936 (34.27)	1574 (34.17)	0.028
Moderate	7282 (33.21)	5685 (32.82)	1597 (34.67)	
High	7134 (32.54)	5699 (32.91)	1435 (31.16)	
Current smoking n (%)	4191 (19.11)	3532 (20.39)	659 (14.31)	< 0.001
Current drinking n (%)	3783 (17.25)	3204 (18.50)	579 (12.57)	< 0.001
Napping n (%)	15,643 (71.34)	12,570 (72.58)	3073 (66.72)	< 0.001
BMI (kg/m^2^, mean ± SD)	24.97 ± 3.61	24.96 ± 3.60	25.02 ± 3.64	0.313
Chronic disease n (%)	13,727 (62.61)	10,601 (61.21)	3126 (67.87)	< 0.001
Utility index (mean ± SD)	0.923 ± 0.113	0.964 ± 0.094	0.909 ± 0.160	< 0.001
VAS scores (mean ± SD)	78.09 ± 14.90	79.69 ± 14.08	72.10 ± 16.33	< 0.001

T-test was performed to compare the differences in continuous variables; Chi-square test was used to compare the differences in the categorical variables

SD, standard deviation; RMB, Renminbi; BMI, Body mass index

### Health problems reported

Table 2 demonstrates the percentage of the five dimensions of the EQ-5D-5L scale according to sleep quality. The most frequently reported problem (23.55%) was reported in the pain/discomfort dimension, followed by the mobility dimension (13.24%), while the least reported (3.84%) was the self-care dimension. The differences between good and poor sleep quality groups in each of the five dimensions were statistical significance (*P* < 0.001). The percentages of the five dimensions of the EQ-5D-5L scale according to night sleep duration are listed in Additional file 1: Table S2. The differences between different night sleep duration groups in each of the five dimensions were statistically significant (*P* < 0.001).

**Table 2:** Reported health problems of respondents according to sleep quality

Dimensions	Total (n = 21,926)	Good (n = 17,320)	Poor (n = 4606)	*P*
*Mobility* n (%)				
No problems	19,022 (86.76)	15,511 (89.56)	3511 (76.23)	< 0.001
Slight problems	2110 (9.62)	1363 (7.87)	747 (16.22)	
Moderate problems	546 (2.49)	308 (1.78)	238 (5.17)	
Severe problems	209 (0.95)	115 (0.66)	94 (2.04)	
Extreme problems	39 (0.18)	23 (0.13)	16 (0.35)	
*Self-care* n (%)				
No problems	21,085 (96.16)	16,876 (97.44)	4209 (91.38)	< 0.001
Slight problems	533 (2.43)	281 (1.62)	252 (5.47)	
Moderate problems	186 (0.85)	96 (0.55)	90 (1.95)	
Severe problems	95 (0.43)	47 (0.27)	48 (1.04)	
Extreme problems	27 (0.12)	20 (0.12)	7 (0.15)	
*Usual activities* n (%)				
No problems	20,450 (93.27)	16,476 (95.13)	3974 (86.28)	< 0.001
Slight problems	993 (4.53)	593 (3.42)	400 (8.68)	
Moderate problems	295 (1.35)	155 (0.89)	140 (3.04)	
Severe problems	130 (0.59)	62 (0.36)	68 (1.48)	
Extreme problems	58 (0.26)	34 (0.20)	24 (0.52)	
*Pain/discomfort* n (%)				
No problems	16,763 (76.45)	13,895 (80.23)	2868 (62.27)	< 0.001
Slight problems	4022 (18.34)	2768 (15.98)	1254 (27.23)	
Moderate problems	860 (3.92)	511 (2.95)	349 (7.58)	
Severe problems	260 (1.19)	134 (0.77)	126 (2.74)	
Extreme problems	21 (0.10)	12 (0.07)	9 (0.20)	
*Anxiety/depression* n (%)				
No problems	20,237 (92.30)	16,316 (94.20)	3921 (85.13)	< 0.001
Slight problems	1265 (5.77)	801 (4.62)	464 (10.07)	
Moderate problems	305 (1.39)	154 (0.89)	151 (3.28)	
Severe problems	100 (0.46)	39 (0.23)	61 (1.32)	
Extreme problems	19 (0.09)	10 (0.06)	9 (0.20)	

Chi-square test was used to compare the differences

### Relationship between sleep quality and night sleep duration and HRQoL

Table 3 summarizes the results of Tobit regression and GLM analyses on the utility index and VAS scores. After multiple adjustments, the Tobit regression model and GLM indicated that utility index and VAS score were lower in poor sleepers than good sleepers. The coefficients [95% confidence interval (*CI*)] for poor sleep quality were − 0.124 (− 0.133, − 0.114) and − 6.25 (− 6.71, − 5.78) on utility index and VAS score, respectively. Compared with the reference group (7 h-), participants who slept shorter or longer were associated with a lower utility index and VAS score. For example, individuals who slept < 6 h were 0.043 (95% *CI* − 0.059, − 0.026) lower in utility index and 2.00 lower (95% *CI* − 2.78, − 1.21) in VAS than those who slept 7 h-. Individuals who slept ≥10 h were 0.071 (95% *CI* − 0.095, − 0.049) lower in utility index and 2.52 lower (95% *CI* − 3.66, − 1.39) in VAS than those who slept 7 h-.

**Table 3:** The results of Tobit regression and generalized linear models analyses on utility index and VAS score

	Model 1 (*β* (95% CI))		Model 2 (*β* (95% CI))		Model 3 (*β* (95% CI))	
	Utility index	VAS score	Utility index	VAS score	Utility index	VAS score
Sleep quality						
Poor	− 0.151 (− 0.161, − 0.142)	− 7.59 (− 8.07, − 7.12)	− 0.130 (− 0.139, − 0.120)	− 6.69 (− 7.16, − 6.23)	− 0.124 (− 0.133, − 0.114)	− 6.25 (− 6.71, − 5.78)
Good	Ref	Ref	Ref	Ref	Ref	Ref
Night sleep duration						
< 6 h	− 0.053 (− 0.070, − 0.036)	− 2.35 (− 3.17, − 1.54)	− 0.045 (− 0.062, − 0.028)	− 2.14 (− 2.94, − 1.35)	− 0.043 (− 0.059, − 0.026)	− 2.00 (− 2.78, − 1.21)
6 h-	− 0.020 (− 0.032, − 0.008)	− 0.50 (− 1.06, 0.06)	− 0.020 (− 0.031, − 0.008)	− 0.56 (− 1.11, − 0.02)	− 0.020 (− 0.032, − 0.009)	− 0.58 (− 1.11, − 0.04)
7 h-	Ref	Ref	Ref	Ref	Ref	Ref
8 h-	− 0.019 (− 0.030, − 0.008)	− 0.29 (− 0.80, 0.21)	− 0.007 (− 0.017, 0.004)	0.19 (− 0.30, 0.68)	− 0.005 (− 0.016, 0.009)	0.26 (− 0.22, 0.75)
9 h-	− 0.052 (− 0.068, − 0.037)	− 1.13 (− 1.85, − 0.41)	− 0.026 (− 0.041, − 0.011)	0.04 (− 0.67, 0.74)	− 0.023 (− 0.038, − 0.009)	0.15 (− 0.54, 0.84)
≥10 h	− 0.125 (− 0.149, − 0.101)	− 5.19 (− 6.38, − 4.01)	− 0.076 (− 0.099, − 0.053)	− 2.87 (− 4.03, − 1.71)	− 0.071 (− 0.095, − 0.049)	− 2.52 (− 3.66, − 1.39)

Model 1: Unadjusted

Model 2: Adjusted age, gender, marital status, education level, average monthly income, physical activity, smoking status, drinking status and napping

Model 3: Adjusted common chronic diseases based on model 2 (including hypertension, dyslipidemia, T2DM, CHD and stroke)

CI, confidence interval; Ref., reference

**Fig. 1:**
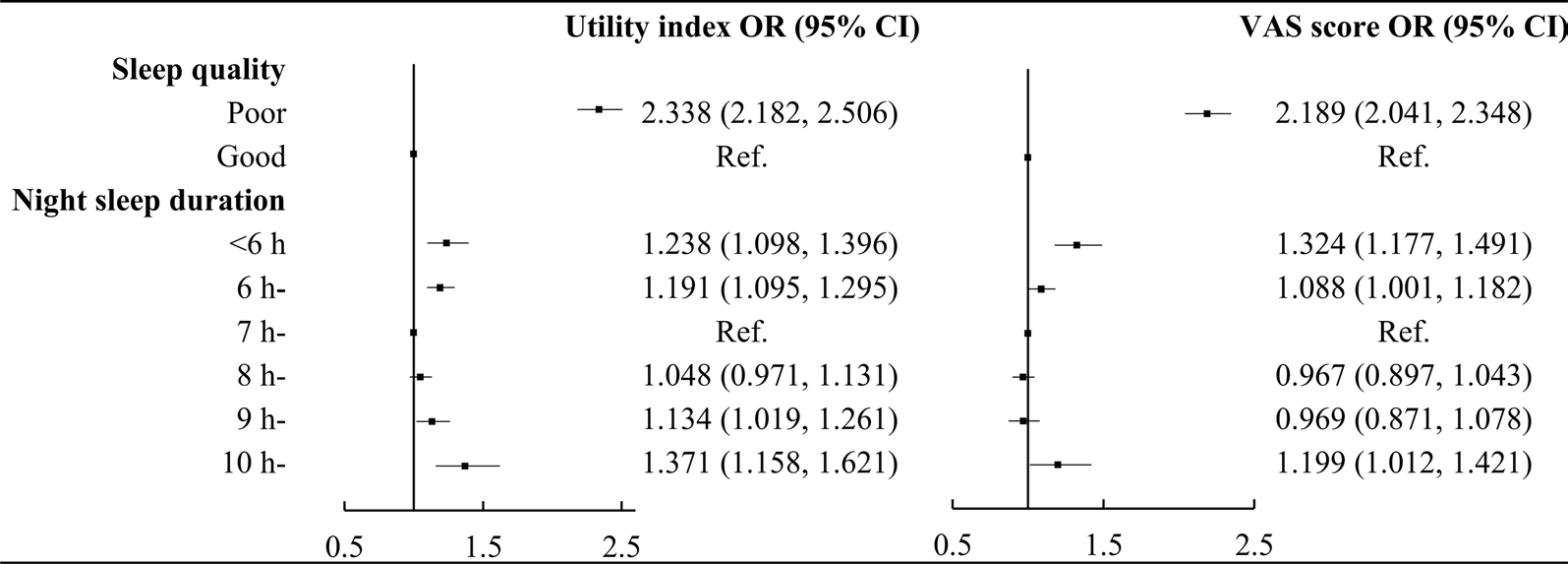
The results of logistic regression on low utility index and VAS score (adjusted age, gender, marital status, education level, average monthly income, physical activity, smoking status, drinking status, napping and common chronic diseases including hypertension, dyslipidemia, T2DM, CHD and stroke)

The results of logistic regression are shown in Fig. 1. Compared with participants with good sleep quality, those with poor sleep quality were more likely to have a low utility index and VAS score (odds ratio (OR) (95% *CI*): 2.338 (2.182, 2.506) in utility index, 2.189 (2.041, 2.348) in VAS score). Similar to the Tobit regression and GLM, the results of logistic regression also indicated that individuals with extreme night sleep duration (both short and long) had a low utility index and VAS score.

**Fig. 2:**
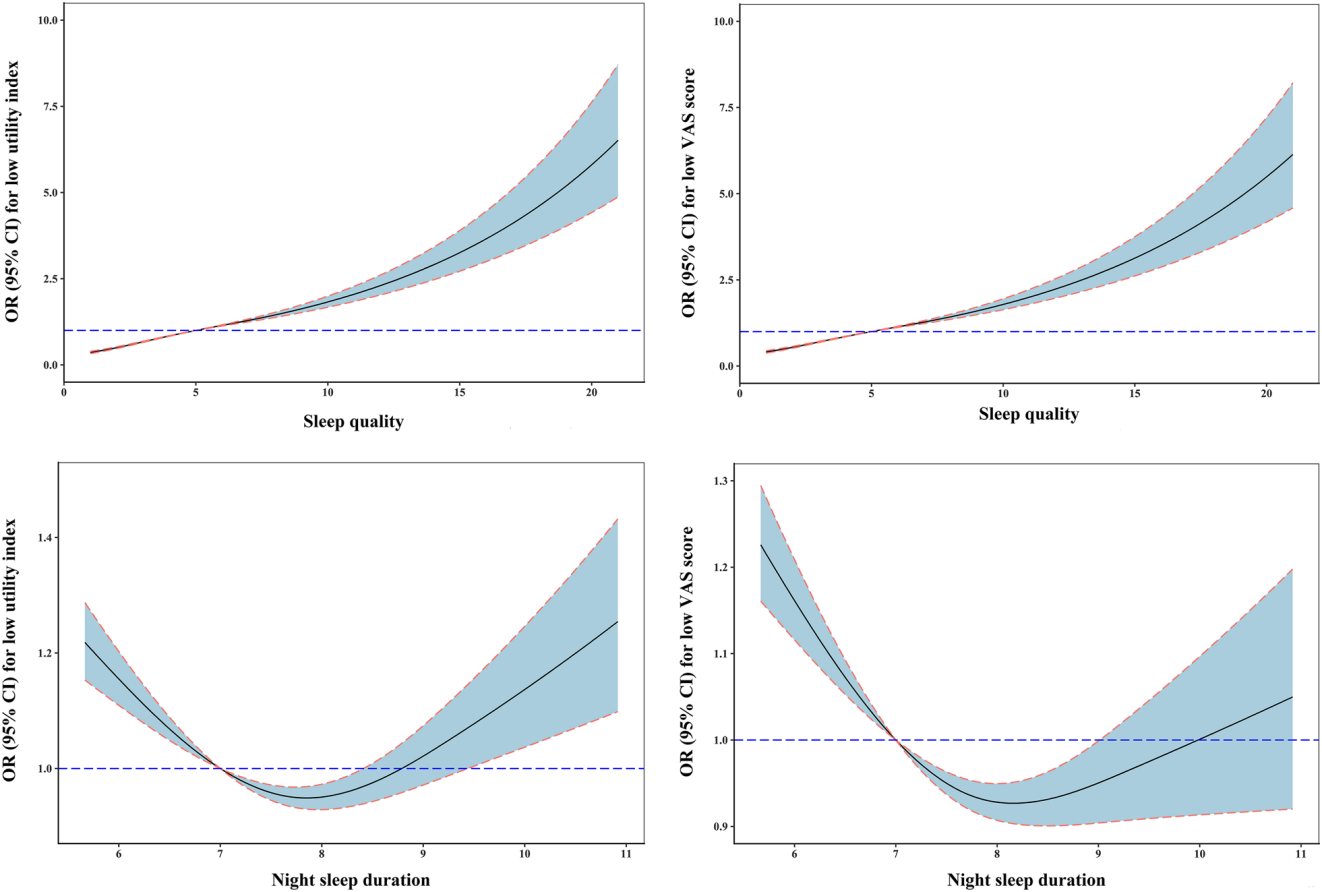
The dose–response relationships of sleep quality and night sleep duration with low utility index and VAS score

In addition, the dose–response relationships between sleep quality and night sleep duration and HRQoL investigated by restricted cubic spline are presented in Fig. 2. A U-shape relationship between night sleep duration and low HRQoL, along with a J-shape relationship between sleep quality and low HRQoL, was observed (*P* for non-linear < 0.001).

### The combined effects of sleep quality and night sleep duration on HRQoL

**Fig. 3:**
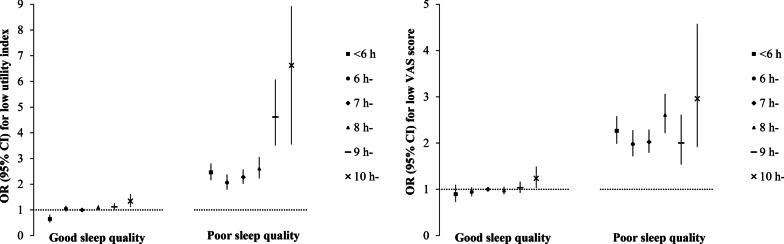
The combined effect of sleep quality and night sleep duration on HRQoL

The combined effects of sleep quality and night sleep duration on HRQoL are shown in Fig. 3 and Additional file 1: Table S3. Compared with participants with good sleep quality and appropriate night sleep duration (7 h-), those with poor sleep quality and long night sleep duration (≥10 h) were strongly associated with a lower utility index and VAS score (OR (95% *CI*): 6.626 (3.548, 8.920) in utility index, 2.962 (1.916, 4.578) in VAS score). The OR (95% *CI*) for individuals with poor sleep quality and short night sleep duration (< 6 h) were 2.459 (2.158, 2.803) and 2.265 (1.985, 2.582) in utility index and VAS score, respectively. Notably, there was no significant association between short sleep duration and low HRQoL in these participants with good sleep quality, while there was a robust association between night sleep duration and low HRQoL in those with poor sleep quality. In the good sleep quality group, even people with long night sleep duration (≥10 h) were only slightly associated with low HRQoL.

## Discussion

The current study investigated the independent and combined effects of sleep quality and night sleep quality on HRQoL. The results indicated that poor sleep quality and extreme night sleep duration (both short and long) were independently associated with low HRQoL. A U-shape relationship between night sleep duration and HRQoL was observed, along with a J-shape relationship between sleep quality and HRQoL. Additionally, the combined effects of sleep quality and night sleep duration on HRQoL were found. Individuals with poor sleep quality and night sleep duration ≥ 10 h were strongly associated with low HRQoL. More importantly, there was no significant association between short sleep duration and low HRQoL in participants with good sleep quality, while there was a robust association between night sleep duration and low HRQoL in those with poor sleep quality.

In this study, most participants were satisfied with their HRQoL, consistent with the previous studies conducted in China [31, 32]. It may be due to the participants of this study were recruited from rural areas and lived a normal life at home, which hinted that HRQOL is better than those who live in medical institutions for professional care. In the five dimensions of EQ-5D-5L, the pain/discomfort dimension was the most prevalent reported problem (23.55%), while the self-care dimension was the least reported problem (3.84%). It was consistent with previous researches in China [12, 13]. This study also found that the prevalence of poor sleep quality was 21.00%, according with previous studies in China [33, 34]. However, this was contrary to a study in Hong Kong [16], which found that only 22.3% of participants had a good sleep quality. This was partially explained by the fact that most of the individuals in this study were women (84.4%) who were more likely to suffer from sleep disturbance [35].

The current study found that poor sleep quality was independently associated with low HRQoL, consistent with two prior studies conducted in Suzhou and Hong Kong [16, 17]. However, the current study was slightly different from these two studies. Firstly, both studies were carried out in the elderly, and they may not fully reflect the relationship between sleep quality and health-related quality of life. Secondly, the sleep quality of the study conducted in Suzhou was assessed by a single question rather than a validity instrument Pittsburgh Sleep Quality Index (PSQI), which may lead to bias. In addition, the study in Hong Kong assessed the HRQoL by 36-Item Short Form (SF-36) questionnaire. Although the utility score calculated from SF-36 was more discriminative than the utility score calculated from EQ-5D-5L [36, 37], no evidence indicated that SF-36 was better than EQ-5D among the general population [25].

This study also found that extreme night sleep duration (both short and long) was independently associated with low HRQoL. The results of previous studies investigating the association between sleep duration and HRQoL were mixed. A previous study among US adults aged ≥ 18 years indicated a positive association between short and long sleep duration and fair/poor self-rated health [38]. Additionally, a study among young adults also found a U-shape relationship between sleep duration and HRQoL [39]. In contrast, another study among university students aged 17–30 years reported that only a short sleep duration was associated with self-rated poor health [40]. Nevertheless, a study found that neither subjective nor act graphic sleep duration was associated with well-being quality [41]. Most of these previous studies focused on sleep duration in 24 h, while the current study was concentrated on night sleep duration. It was a benefit for the establishment of a healthy sleep habit.

To the best of our knowledge, this is the first study to investigate the combined effect of sleep quality and night sleep duration on HRQoL. The results indicated that individuals with poor sleep quality and long sleep duration (≥ 10 h) were strongly associated with low HRQoL. The mechanisms underlying the associations between sleep quality and night sleep duration and HRQoL remain unclear. It may be explained by poor sleep quality and extreme night sleep duration can induce some chronic conditions. Previous studies have reported that poor sleep quality and extreme night sleep duration were associated with broad-spectrum chronic conditions, such as cognitive impairment, depressive and anxiety symptoms, hypertension, and CHD [5–8, 42, 43]. Nonetheless, it should be noted that our findings based on a cross-sectional study cannot confirm the causal relationship between sleep quality and night sleep duration and HRQoL. Certainly, a study suggested an inverse cause-effect relationship between sleep and health [44]. This study also found that there was no significant association between short sleep duration and low HRQoL in the good sleep quality group, while there was a robust association between night sleep duration and worse HRQoL in the poor sleep quality group. This finding indicated that perceived sleep quality was a critical moderating factor in the relationship between night sleep duration and HRQoL, consistent with previous work by *Lichstein KL* [45].

There are several limitations in this study. Firstly, the current study was a cross-sectional design, which was limited to identifying the causal relationship between sleep quality and night sleep duration and HRQoL. Secondly, the results were based on only a province of China, which might not be a representative sample of the Chinese rural population. However, the rural population of Henan province accounts for 8.9% of the rural Chinese population, and the results based on this relatively large rural cohort study, to some extent, could represent the Chinese rural population. Thirdly, some information of participants in this study was collected based on self-reported, but higher test–retest reliability, effective training of study workers, and good field implementation will ensure the accuracy and reliability of the information. In addition, we neglect the cause of the poor sleep quality, extreme night sleep duration, and use of sleep medication, which may induce bias.

## Conclusion

In this study, a U-shape relationship between night sleep duration and HRQoL was observed, along with a J-shape relationship between sleep quality and HRQoL. In addition, individuals with longer night sleep duration (≥10 h) and poorer sleep quality were strongly associated with low HRQoL. These results suggested that maintaining a good sleep quality and appropriate night sleep duration may be an effective way to improve the HRQoL for individuals in rural China.

## Supplementary Information

**Additional file 1: Supplementary table 1.** Characteristics of study participants according to night sleep duration. **Supplementary table 2**. Reported health problems of respondents according to night sleep duration. **Supplementary table 3**. The combined effect of sleep quality and night sleep quality on HRQoL.

## Data Availability

The data analyzed during current study are available from the corresponding author on reasonable request.

## Abbreviations

HRQoL: Health-related quality of life
EQ-5D-5L: European Quality of Life Five Dimension Five Level Scale
PSQI: Pittsburgh Sleep Quality Index
GLM: Generalized linear model
CI: Confidence interval
OR: Odds ratio
CHD: Coronary heart diseases
T2DM: Type 2 diabetes mellitus
BMI: Body mass index
IPAQ: Physical Activity Questionnaire
MO: Mobility
SC: Self-care
UA: Usual activities
PD: Pain/discomfort
AD: Anxiety/depression
VAS: Visual Analogue Scale
SD: Standard deviation
