# Supplementary material for: Independent and combined effects of sleep quality and night sleep duration on health-related quality of life in rural areas: a large-scale cross-sectional study
Source: Health Qual Life Outcomes. 2022 Feb 21;20:31. doi: 10.1186/s12955-022-01936-8 (PMC8862521; doi:10.1186/s12955-022-01936-8)
Supplement: Supplementary file 1 — Additional file 1: Supplementary table 1. Characteristics of study participants according to night sleep duration. Supplementary table 2. Reported health problems of respondents according to night sleep duration. Supplementary table 3. The combined effect of sleep quality and night sleep quality on HRQoL. [file 12955_2022_1936_MOESM1_ESM.docx]

**Supplementary table 1.** **Characteristics of study participants according to night sleep duration.**

| **Variable** | **<6 h (n=1519)** | **6~ h (n=4102)** | **7~ h (n=7793)** | **8~ h (n=5766)** | **9~ h (n=2082)** | **≥ 10 h (n=658)** | ***P*** |
| --- | --- | --- | --- | --- | --- | --- | --- |
| Age (year, mean ± SD) | 56.41 ± 10.97 | 55.12 ± 11.45 | 54.94 ± 12.13 | 56.01 ± 13.20 | 58.59 ± 13.26 | 61.85 ± 12.65 | <0.001 |
| Women n (%) | 890 (58.59) | 2430 (59.24) | 4664 (59.85) | 3614 (52.68) | 1284 (61.67) | 385 (58.51) | 0.001 |
| Educational level n (%) |  |  |  |  |  |  |  |
| Elementary school or below | 686 (45.16) | 1641 (40.00) | 3162 (40.57) | 2714 (47.07) | 1194 (57.35) | 436 (66.26) | <0.001 |
| Junior high school | 582 (38.82) | 1640 (39.98) | 3091 (39.66) | 2099 (36.40) | 613 (29.44) | 163 (24.77) |  |
| Senior high school or above | 251 (16.52) | 821 (20.02) | 1540(19.77) | 935 (16.53) | 275 (13.21) | 59 (8.97) |  |
| Marital status n (%) |  |  |  |  |  |  |  |
| Married/cohabiting | 1341 (88.28) | 3717 (90.61) | 7108 (91.21) | 5158 (89.46) | 1846 (88.66) | 538 (81.76) | <0.001 |
| Widowed/separated/divorced | 162 (10.66) | 321 (7.83) | 567 (7.28) | 504 (8.74) | 204 (9.80) | 98 (14.89) |  |
| Single | 16 (1.05) | 64 (1.56) | 118 (1.51) | 104 (1.80) | 32 (1.54) | 22 (3.34) |  |
| Average monthly income n (%) |  |  |  |  |  |  |  |
| <500 RMB | 565 (37.20) | 1479 (36.06) | 2825 (36.25) | 2280 (39.54) | 880 (42.27) | 346 (52.58) | <0.001 |
| 500- RMB | 477 (31.40) | 1337 (32.59) | 2503 (32.12) | 1803 (31.27) | 658 (31.60) | 179 (27.20) |  |
| ≥1000 RMB | 477 (31.40) | 1286 (31.35) | 2465 (31.63) | 1683 (29.19) | 544 (26.13) | 133 (20.21) |  |
| Physical activity n (%) |  |  |  |  |  |  |  |
| Low | 489 (32.19) | 1322 (32.23) | 2643 (33.92) | 2008 (34.82) | 752 (36.12) | 293 (44.53) | <0.001 |
| Moderate | 492 (32.39) | 1342 (32.72) | 2669 (34.25) | 1946 (33.75) | 664 (31.89) | 167 (25.38) |  |
| High | 538 (35.42) | 1438 (35.06) | 2481 (31.84) | 1812 (31.43) | 666 (31.99) | 198 (30.09) |  |
| Current smoking n (%) | 394 (25.94) | 904 (22.04) | 1502 (19.27) | 944 (16.37) | 350 (16.81) | 97 (14.74) | <0.001 |
| Current drinking n (%) | 329 (21.66) | 787 (19.19) | 1375 (17.64) | 878 (15.23) | 312 (14.99) | 102 (15.50) | <0.001 |
| Napping n (%) | 1016 (66.89) | 2997 (73.06) | 5753 (73.82) | 4079 (70.74) | 1380 (66.28) | 414 (62.92) | <0.001 |
| BMI (kg/m2, mean ± SD) | 25.29 ± 3.65 | 25.05 ± 3.62 | 24.98 ± 3.51 | 24.86 ± 3.61 | 24.87 ± 3.81 | 24.82 ± 3.78 | <0.001 |
| Chronic disease n (%) | 984 (64.78) | 2476 (60.36) | 4774 (61.26) | 3640 (63.13) | 1375 (66.04) | 474 (72.04) | <0.001 |
| Utility index (mean ± SD) | 0.940 ± 0.140 | 0.957 ± 0.097 | 0.960 ± 0.098 | 0.956 ± 0.104 | 0.941 ± 0.132 | 0.906 ± 0.186 | <0.001 |
| VAS scores (mean ± SD) | 76.34 ± 15.78 | 78.18 ± 14.50 | 78.71 ± 14.38 | 78.44 ± 14.66 | 77.59 ± 15.87 | 73.54 ± 18.37 | <0.001 |

Abbreviation: SD, standard deviation; RMB, Renminbi; BMI, Body mass index.

Kruskal-Wallis H test was performed to compare the differences in continuous variables; Chi-square test was used to compare the differences in the categorical variables.

**Supplementary table 2. Reported health problems of respondents according to night sleep duration.**

| **Variable** | **<6 h (n=1519)** | **6~ h (n=4102)** | **7~ h (n=7793)** | **8~ h (n=5766)** | **9~ h (n=2082)** | **≥ 10 h (n=658)** | ***P*** |
| --- | --- | --- | --- | --- | --- | --- | --- |
| **Mobility** n (%) |  |  |  |  |  |  |  |
| No problems | 1282 (84.40) | 3625 (88.37) | 6891 (88.43) | 5003 (86.77) | 1714 (82.32) | 502 (76.29) | <0.001 |
| Slight problems | 170 (11.19) | 356 (8.68) | 673 (8.64) | 554 (9.61) | 264 (12.68) | 92 (13.98) |  |
| Moderate problems | 41 (2.70) | 93 (2.27) | 160 (2.05) | 145 (2.51) | 68 (3.27) | 39 (5.93) |  |
| Severe problems | 22 (1.45) | 26 (0.63) | 57 (0.73) | 55 (0.95) | 29 (1.39) | 20 (3.04) |  |
| Extreme problems | 4 (0.26) | 2 (0.05) | 12 (0.15) | 9 (0.16) | 7 (0.34) | 5 (0.02) |  |
| **Self-care** n (%) |  |  |  |  |  |  |  |
| No problems | 1440 (94.80) | 3962 (96.59) | 7564 (97.06) | 5563 (96.48) | 1962 (94.24) | 588 (89.36) | <0.001 |
| Slight problems | 52 (3.42) | 107 (2.61) | 144 (1.85) | 119 (2.06) | 76 (3.65) | 35 (5.32) |  |
| Moderate problems | 17 (1.12) | 18 (0.44) | 58 (0.74) | 46 (0.80) | 29 (1.39) | 18 (2.74) |  |
| Severe problems | 9 (0.53) | 14 (0.34) | 23 (0.30) | 26 (0.45) | 13 (0.62) | 11 (1.67) |  |
| Extreme problems | 2 (0.13) | 1 (0.02) | 4 (0.05) | 12 (0.21) | 2 (0.10) | 6 (0.91) |  |
| **Usual activities** n (%) |  |  |  |  |  |  |  |
| No problems | 1397 (91.97) | 3863 (94.17) | 7358 (94.42) | 5399 (93.64) | 1882 (90.39) | 545 (82.83) | <0.001 |
| Slight problems | 81 (5.33) | 183 (4.46) | 296 (3.80) | 240 (4.16) | 128 (6.15) | 65 (9.88) |  |
| Moderate problems | 21 (1.38) | 36 (0.88) | 91 (1.17) | 74 (1.28) | 46 (2.21) | 27 (4.10) |  |
| Severe problems | 15 (0.99) | 14 (0.34) | 34 (0.44) | 36 (0.62) | 18 (0.86) | 13 (1.98) |  |
| Extreme problems | 5 (0.33) | 6 (0.15) | 14 (0.18) | 17 (0.29) | 8 (0.38) | 8 (1.22) |  |
| **Pain/discomfort** n (%) |  |  |  |  |  |  |  |
| No problems | 1095 (72.09) | 3101 (75.60) | 6143 (78.83) | 4424 (76.73) | 1541 (74.02) | 453 (68.84) | <0.001 |
| Slight problems | 321 (21.13) | 800 (19.50) | 1309 (16.08) | 1055 (18.30) | 401 (19.26) | 136 (20.67) |  |
| Moderate problems | 63 (4.15) | 152 (3.71) | 274 (3.52) | 221 (3.83) | 105 (5.04) | 45 (6.84) |  |
| Severe problems | 36 (2.37) | 46 (1.12) | 64 (0.82) | 58 (1.01) | 33 (1.59) | 23 (3.50) |  |
| Extreme problems | 4 (0.26) | 3 (0.07) | 3 (0.04) | 8 (0.14) | 2 (0.10) | 1 (0.15) |  |
| **Anxiety/depression** n (%) |  |  |  |  |  |  |  |
| No problems | 1379 (90.78) | 3752 (91.47) | 7246 (92.98) | 5337 (92.56) | 1927 (92.56) | 591 (89.82) | <0.001 |
| Slight problems | 89 (5.86) | 259 (6.31) | 419 (5.38) | 341 (5.91) | 112 (5.38) | 44 (6.69) |  |
| Moderate problems | 33 (2.17) | 66 (1.61) | 97 (1.24) | 65 (1.13) | 26 (1.25) | 18 (2.74) |  |
| Severe problems | 16 (1.05) | 22 (0.54) | 23 (0.30) | 20 (0.35) | 14 (0.67) | 5 (0.76) |  |
| Extreme problems | 2 (0.13) | 3 (0.07) | 8 (0.10) | 3 (0.05) | 3 (0.14) | 0 (0.00) |  |

Chi-square test was used to compare the differences.

**Supplementary table 3. The combined effect of sleep quality and night sleep quality on HRQoL.**

| Night sleep quality | Utility index | |  | VAS score | |
| --- | --- | --- | --- | --- | --- |
|  | Sleep quality | |  | Sleep quality | |
|  | Good | Poor |  | Good | Poor |
| <6 h | 0.645 (0.510, 0.815) | 2.459 (2.158, 2.803) |  | 0.895 (0.727, 1.100) | 2.265 (1.985, 2.582) |
| 6~ h | 1.043 (0.940, 1.158) | 2.061 (1.789, 2.375) |  | 0.943 (0.851, 1.045) | 1.977 (1.715, 2.279) |
| 7~ h | Ref. | 2.275 (2.012, 2.571) |  | Ref. | 2.026 (1.791, 2.291) |
| 8~ h | 1.089 (1.001, 1.185) | 2.608 (2.226, 3.055) |  | 0.972 (0.894, 1.056) | 2.606 (2.216, 3.064) |
| 9~ h | 1.123 (0.999, 1.263) | 4.616 (3.508, 6.074) |  | 1.037 (0.924, 1.165) | 2.004 (1.536, 2.613) |
| 10~ h | 1.343 (1.116, 1.617) | 6.626 (3.548, 8.920) |  | 1.240 (1.030, 1.492) | 2.962 (1.916, 4.578) |
